# Factors associated with utilization of modern postpartum family planing methods during the extended postpartum period among mothers who gave birth in the last 12 months at Injibara town, Northwest, Ethiopia: a cross-sectional study

**DOI:** 10.1186/s40834-022-00191-y

**Published:** 2022-12-01

**Authors:** Getachew Andualem, Almaz Aklilu, Getahun Belay, Wondu Feyisa, Fentahun Alemnew

**Affiliations:** 1 Bahir Dar Health Sciences College, Bahir Dar City, Ethiopia; 2 Department of Midwifery, College of Medicine and Health Sciences, Bahir Dar University, P.Box:079, Bahir Dar City, Ethiopia

**Keywords:** Extended postpartum period, Postpartum, Modern family planning methods, Utilization

## Abstract

**Background:**

The extended postpartum period is the first twelve months following childbirth and is an important entry point for family planning service providers to reduce unintended and too closely spaced pregnancies. A modern postpartum family planning service is one of the recommended public health interventions for reducing maternal and child morbidity and mortalities in sitting where maternal mortality is high, like in Ethiopia.

**Objective:**

This study was aimed to assess factors associated with the utilization of modern family planning methods during the extended postpartum period among mothers who gave birth in the last 12 months at Injibara town, Northwest, Ethiopia.

**Methods:**

A community-based cross-sectional study design was employed from March 1–15/2019 at Injibara town among 402 mothers. The data was collected by a simple random sampling technique and analyzed using the SPSS 23.0 version. Logistic regression analyses were employed to estimate the crude and adjusted odds ratio with a confidence interval of 95% and a *P*-value of < 0.05 in multivariable analysis considered statistically significant. Frequency tables and descriptive summaries were used to describe the study variables.

**Results:**

The total sample size of this study was 402 and among them, 400 postpartum mothers participated in the study with a response rate of 99.5%. The utilization of modern family planning methods during the extended postpartum period among postpartum mothers was 58.5% [95% CI: 53.5- 63%]. Of these, 38.9% and 32.9% of the mothers were using injectables and implants respectively. Secondary and above educational level, having ≥ 3 antenatal care visits, resumption of menstruation, discussing with the partner on utilization of family planning method, being counseled on family planning method utilization during antenatal care visits and immediately after delivery, linkage of the mothers to a family planning unit during child immunization, and having good knowledge of family planning methods were associated with utilization of modern family planning methods during the extended postpartum period.

**Conclusion:**

The utilization of modern family planning methods during the extended postpartum period among postpartum women was low compared to the world health organization recommendation. Socio-demographic, health care service uptakes, and reproductive characteristics were associated with the utilization of modern family planning methods during the extended postpartum period. We suggest emphasizing the education and counseling of women on the utilization of family planning during maternal and child health care service utilization. Mothers should be encouraged to start using modern family planning methods before the resumption of menses.

## Introduction

The extended postpartum period is the first 12 months following childbirth, which is the critical period for addressing many routine interventions, including the provision of family planning (FP) methods [1]. FP is defined as the ability of an individual and couple to attain their desired number of children in a family, the age interval between children, and the timing of their births through the use of family planning methods [2]. Postpartum family planning (PPFP) helps to prevent unintended and closely spaced pregnancies through the first 12 months following childbirth [3].

Worldwide, more than 90% of women during the postpartum period want to either delay or avoid future pregnancies. However, in most cases, sexual activity is resumed without using any FP method [4]. Pregnancies that are either too early, close, or late, expose mothers to high morbidity and mortality during pregnancy and childbirth, and it also has adverse outcomes for the baby [5–7]. When a mother becomes pregnant early after childbirth, she is more likely to develop complications including, spontaneous abortion, postpartum bleeding, anemia, low birth weight newborn, and preterm delivery. The earlier delivered child may not receive adequate care and support which, thereafter, could lead to vulnerabilities to disease and malnutrition [8].

According to the analysis done in 172 countries, without FP method use, the number of maternal deaths would have been 1.8 times higher than with FP method use, which means that FP method use averted 44.3% of maternal deaths [9]. In Ethiopia, the maternal mortality is 412 per 100, 000 live births [10]. Providing PPFP can reduce unintended pregnancies, maternal and fetal morbidity and mortality, and unsafe abortions by reducing the proportion of births at high risk [5, 11]. PPFP has the potential to reduce 71% of unwanted pregnancies: abolishing 53 million unintended pregnancies, 22 million unplanned births, 25 million induced abortions, and 7 million miscarriages [12].

According to the World Health Organization (WHO) recommendations, after a woman has given birth, she should stay for at least two years before the next pregnancy to reduce the risk of adverse maternal and infant outcomes [1]. The recommended time for the initiation of the FP method in the postpartum period is 6 weeks after delivery, but most women do not start taking the FP method at the recommended time [4]. The first years after delivery is an important entry point for FP service provision. However, evidence has shown that 47% of pregnancies occur within a short birth interval or less than two years after the preceding birth [13].

In Ethiopia, FP services are integrated into maternal and child health care services at all levels of the health care delivery system [14]. Efforts made by the Ethiopian government health policy to strengthen maternal and child health morbidity and mortality reduction strategies like the different reproductive health services and health education being given by health workers, strengthening women's development army, and community health insurance [15]. In Ethiopia, the utilization of PPFP ranges from 10.3% to 80.3% [16, 17]. Reports of two different systematic reviews and meta-analysis studies done in Ethiopia show that the pooled magnitude of the utilization of modern PPFP methods was 45.79% and 48.11% [18, 19].

Women are more likely to engage with the healthcare system during antenatal care (ANC), delivery, postnatal care (PNC), and infant immunizations. Each of these encounters is an opportunity for health care workers to integrate FP into their existing counseling and services to better meet the needs of postpartum women [1]. Modern FP methods for couples are essential to prevent pregnancy-related health risks in women, reduce infant mortality, empower women, enhance education, reduce adolescent pregnancies, and slow population growth [4, 11]. The postpartum period is an ideal and important window of opportunity to initiate an effective FP method and addresses the unmet need for FP and space pregnancy [20]. However, most of the time, women did not start to use modern PPFP methods at the recommended time for the initiation of FP methods, particularly in the study area. Therefore, this study was aimed to assess the prevalence of utilization of modern PPFP methods during the extended postpartum period and its associated factors in Injibara town, Northwest, Ethiopia.

## Methods

### Study design and period

A community-based cross-sectional study was done from March 01–15/2019 at Injibara town.

### Study setting

The study was conducted at Injibara town, Awi zone, Northwest, Ethiopia. Injibara town is located about 447 km away from Addis Ababa, the capital city of Ethiopia, and 118 km from Bahir Dar city in the Amhara Region. The town has five Keeble’s (the lowest administrative unit in Ethiopia, next to the district) with a total population of 46,745, of which 23,466 are females, and women of reproductive age were about 11,048 and 1,578 mothers who were delivered in the preceding year. There are one general hospital, one public health center, and five health posts in the town, which provide maternal and other health services to the population of the town and the surrounding area [21].

### Source population

All mothers who gave birth in the last 12 months in Injibara Town.

### Study population

All randomly selected mothers who gave birth in the last 12 months at Injibara Town.

### Inclusion and exclusion criteria

Mothers who gave birth in the last 12 months and lived in the area at least for six months preceding the survey regardless of their birth outcome were included in the study, while mothers who had a history of intrapartum hysterectomy were excluded.

### Sample size determination

The sample size was calculated using a single population proportion formula by considering the following assumptions: the utilization of modern PPFP methods in Gondar town (45.8%) [22], Zα/2 = critical value for normal distribution at 95% confidence level, which is equal to 1.96 (Z value of alpha = 0.05), and a 5% margin of error (ω = 0.05).

$$\mathrm{Sample}\;\mathrm{size}\;(\mathrm n)=\;\frac{{(\mathrm{Z}\alpha/2)}^2\;\mathrm p\;(1-\mathrm p)\;}{\mathrm d^2},\;\mathrm n=\;\frac{{(1.96)}^2\;0.458\;(1-0.458)\;}{{(0.05)}^2}=\;382$$

The sample size was adjusted by adding a 5% non-response rate and the final sample size was 402 postpartum mothers.

### Sampling procedure

All the five kebeles of Injibara town were included in the study. The total sample size was proportionally allocated for each kebele of the town based on the total number of postpartum mothers. Before data collection, a census was conducted to identify the total numbers of postpartum women in each kebele of the town. After the census, the total number of postpartum mothers in the town was 1,578. The numbers of postpartum mothers in kebele 1, 2, 3, and 4 were 354, 410, 84, and 169 respectively. The total sample size was proportionally allocated for each kebele of the town, based on their population size.

After proportional allocation, the sample size was 95, 104, 21, and 43 for kebele 1, 2, 3, and 4 respectively. We used a simple random sampling technique and the study participants were selected by a lottery method. An attempt was made three times to interview the respondent and after all, they were considered non-responders.

Dependent variable: Utilization of modern PPFP methods during the extended postpartum period.

Independent variables include socioeconomic and demographic factors (age of the mothers, marital status, educational status of mothers and partner, religion, ethnicity, occupation of mothers and partner, and average monthly income of the family), reproductive and health service utilization-related factors (parity, number of alive children, birth interval, history of abortion, ANC visit, previous utilization of FP methods, place of delivery, PNC check-up, postnatal period, counseling on utilization of FP method during pregnancy and after delivery, discussed on utilization of PPFP method with a partner, immunization visits, getting linked to FP unit during child immunization, the timing of starting FP method utilization, types of FP method used, sexual and menstrual resumption after delivery), knowledge, source of information and attitude towards FP methods.

### Operational definitions

Extended postpartum period—The time from childbirth up to one year of delivery [4, 23].

Postpartum modern contraceptive utilization – A postpartum woman's current use of any modern FP methods (oral pills, IUCD, injectable, condom (male or female), sterilization (male or female), implants) during the 12 months following her index childbirth. The utilization was measured by mothers’ words with yes or no options for use (yes = 1, no = 0) [3, 24, 25].

Knowledge: In this study, refer to the knowledge of mothers about modern FP methods. It was evaluated by the mother's answer to the knowledge-related questions. Mother was considered to have good knowledge if she correctly answered greater than or equal to the mean score of the total knowledge assessing questions [22, 26].

Attitude: In this study, it refers to the attitude of mothers toward modern FP methods. It was evaluated by the mother's answer to the attitude-related questions. Mother was considered to have a favorable attitude if she correctly answered greater than or equal to the mean score of the total attitude assessing questions [22, 26].

### Data collection tools and procedures

A structured interviewer-administered questionnaire was used to collect the data, which was adapted from relevant works of literature and modified to the local context [22, 27–29]. The questionnaire was first prepared in the English language, then it was translated into Awigni by an individual who has a good ability in these languages, then retranslated back into English to check the consistency. The questionnaire consisted of socio-demographic characteristics, obstetrics and reproductive history, maternal health care, knowledge and attitude towards modern FP methods, current utilization of modern PPFP methods, previous experience of FP services, and sexuality issue-related variables. Knowledge-related questions were assessed by + 1 for correct answers and 0 for incorrect answers. Attitude-related questions were assessed by assigning + 1 for strongly disagree, + 2 for disagree, + 3 for neutral, + 4 for agree, and + 5 for strongly agree. Then the score for each mother was summed up and categorized. A pre-tested structured interviewer-administered questionnaire was used for data collection purposes. The data was collected by five-diploma midwives and supervised by two BSc midwives.

### Data quality control

Data were collected by trained data collectors and pretesting of the instrument was done before the actual data collection. The questionnaire was pre-tested before the actual data collection period on 5% (20) mothers nearer to Injibara town, at Addis Kidam town, which has similar characteristics to the study population, to ensure the clarity of the questionnaire, to check the wording, and to confirm the logical sequence of the questions. Data collectors and the supervisors were trained for two days by the investigators. After necessary modifications and corrections were done to standardize and ensure its reliability and validity, additional adjustments were made based on the results of the pre-test. The completeness of the data was checked by data collectors during data collection and daily supervision was done for data completeness.

### Data processing and analysis

The data were entered into Epi data 3.1, edited and cleaned for inconsistencies, missing values, and outliers, then exported to SPSS version 23.0 for analysis. During the analysis, all explanatory variables which have a significant association in bivariate analysis with a *P*-value < 0.20 were entered into a multivariable logistic regression model to get an AOR, and those variables with 95% of CI and a *P*-value of < 0.05 in the multivariable analysis was considered as statistically significant with the utilization of modern PPFP methods. The multicollinearity test was done using the variance inflation factor and there was no collinearity existing between the independent variables. The model goodness of the test was checked using the Hosmer- Lemeshow goodness of the fit and its *P*-value was 0.215. Frequency tables and descriptive summaries were used to describe the study variables.

## Results

### Socio-demographic characteristics

The total sample size of this study was 402 and among them, 400 postpartum mothers participated in our study with a response rate of 99.5%. The mean age of the mothers was 26.82 (± 4.87 SD), ranging from 18–40 years. Of these, 166 (41.5%) were found in the age group of 25–29 years. About 81% (n = 324) were followers of the Orthodox Christianity religion and 381 (95.2%) of the study participants belong to the Amhara ethnicity. In this study, 386 (96.5%) of the mothers were married and 214 (53.3%) were housewives. Of the mothers, 119 (29.8%) had a primary education level and 301 (75.2%) had a monthly family income greater than or equal to 2000 Ethiopia Birr (Table 1).

**Table 1:** Socio-demographic characteristics of the mothers who gave birth in the last 12 months at Injibara town, Northwest Ethiopia, 2019 (*n* = 400)

**Variables**	**N**o**. (%)**
Maternal age in years	
≤ 19	22 (5.5)
20–24	122 (30.5)
25–29	166 (41.5)
30–34	59 (14.8)
≥ 35	31 (7.7)
Marital status	
Married	386 (96.5)
Others^a^	14 (3.5)
Religion	
Orthodox	324 (810)
Muslim	49 (12.2)
Others^b^	27 (6.8)
Ethnicity	
Amhara	381 (95.2)
Others^c^	19 (4.8)
Maternal educational level	
Had no formal education	55 (13.7)
Primary education	119 (29.8)
Secondary education	116 (29.0)
Diploma and above	110 (27.5)
Occupation of the mother	
Housewife	214 (53.3)
Government/NGO employed	79 (19.8)
Merchant	88 (22.0)
Others^d^	19 (4.9)
Husband education (*n* = 386)	
Had no formal education	31 (8.0)
Primary education	73 (19.1)
Secondary education	148 (38.3)
Diploma and above	134 (34.7)
Husband occupation (*n* = 386)	
Government/NGO employed	148 (38.3)
Self-employed	198 (51.3)
Daily laborer	26 (6.8)
Jobseeker	14 (3.6)
Average monthly income of the family	
< 700 Birr	23 (5.8)
700—2000 Birr	76 (19.0)
≥ 2000 Birr	301 (75.2)

^a^Divorce, Widowed and Single

^b^Protestant and Catholic

^c^Oromo and Tigre

^d^Student and Daily laborer

### Reproductive and maternal health service use-related characteristics

In our study, 278 (69.5%) of the mothers were multiparous and 141 (41.0%) had a birth interval of 2–3 years. For their index child, 293 (73.8%) of the mothers had ≥ 3 ANC visits and 213 (53.2%) were counseled on the utilization of modern FP during their ANC visits. Regarding the place of delivery for their index child, 395 (98.7%) gave birth at a health facility and 187 (46.8%) had a history of PNC utilization. About 51.0% (*n* = 197) of mothers discuss the utilization of FP methods with their partner and 218 (54.5%) had a history of previous FP method utilization. Of the mothers, currently, 247 (61.8%) had sexual activity, and 209 (52.2%) mothers' menses were resumed (Table 2).

**Table 2:** Reproductive and maternal health service use-related characteristics of the mothers who gave birth in the last 12 months at Injibara town, Northwest Ethiopia, 2019, (*n* = 400)

**Variables**	**No. (%)**
Parity	
Primipara	122 (30.5)
Multipara	278 (69.5)
Birth interval(*n* = 278)	
< 2 years	52 (18.7)
2 -3 years	114 (41.0)
3–4 years	98 (35.3)
> 4 years	14 (5.0)
Discuss on the use of FP methods with a partner ( n = 386)	
Yes	197 (51.0)
No	189 (49.0)
Previous use of FP methods	
Yes	218 (54.5)
No	182 (45.5)
Number of ANC visit	
Less than three	107 (26.8)
≥ Three	293 (73.8)
Family planning counseling during ANC	
Yes	213 (53.2)
No	187 (46.8)
Previous history of abortion	
Yes	37 (90.7)
No	363 (9.3)
Place of delivery	
Health facility	395 (98.7)
Home	5 (1.3)
PNC checkup	
Yes	187 (46.8)
No	213 (53.2)
Postpartum period(wk)	
≤ 6 week	59 (14.7)
6–12 week	81 (20.3)
13–24 week	114 (28.5)
25–48 week	146 (36.5)
Immunization visit	
Yes	345 (86.2)
No	55 (13.8)
Getting linkage to FP during child immunization (*n* = 345)	
Yes	261 (75.7)
No	84 (24.3)
Menses resumed after recent childbirth	
Yes	209 (52.2)
No	191 (47.8)
Resumed sexual activities after birth	
Yes	247 (61.8)
No	153 (38.2)

### Knowledge and utilization of modern PPFP methods during the extended postpartum period

All study participants had heard about at least one modern contraceptive method, with most obtaining their information from health extension workers (*n* = 357, 89.2%). Of the mothers, 366 (91.5%) heard about injectable FP methods and 265 (66.3%) responded that PPFP should be started, to use within two months of delivery. About 340 (85.0%) of mothers knew that FP methods are used for birth spacing. Based on the predetermined criteria, 248 (62.0%) had good knowledge of FP methods.

In this study, the utilization of modern PPFP methods during the extended postpartum period was 234 (58.5%) [95% CI: 53.5- 63%]. The most commonly used FP method was injectable 91 (38.9%), followed by implants 77 (32.9%). Among current FP methods users, 103 (44%) of women started to use modern PPFP methods within 6 weeks to 3 months of delivery and 149 (63.7%) of postpartum mothers started to use the methods after menses resumed. The major reasons for not currently using the FP method were the feeling of not being exposed to the risk of pregnancy due to amenorrhea after birth 48 (29.0%) and followed by the feeling of not being exposed to the risk of pregnancy due to breastfeeding 36 (22.0%) (Table 3).

**Table 3:** Knowledge and utilization of modern PPFP methods in the extended postpartum period among mothers who gave birth in the last 12 months at Injibara town, Northwest Ethiopia, 2019, (*n* = 400)

Variables	No. (%)
Heard about modern FP methods	
Yes	100.0
Source of information	
Health care providers	288 (72.0)
Health extension workers	357 (89.2)
Mass media	214 (53.5)
Family/friends	104 (26.0)
Which FP methods did you know	
Injectables	366 (91.5)
Oral pills	334 (83.5)
Implants (implanion/jadelle)	266 (66.5)
IUCD	285 (71.3)
Emergency pills	187 (46.8)
Condom	248 (62.0)
Permanent	201 (50.2)
Timing of starting to use PPFP methods	
Within two months of delivery	265 (66.3)
After two months	135 (33.7)
The benefit of FP methods utilization	
Birth spacing	340 (85.0)
Limiting the number of children	281 (70.3)
Prevention of unwanted pregnancy	284 (71.0)
Prevent possible maternal death	222 (55.5)
Prevent STIs	210 (52.5)
Knowledge of modern FP methods	
Good Knowledge	248 (62.0)
Poor Knowledge	152 (38.0)
Current user of modern PPFP methods	
Yes	234 (58.5)
No	166 (41.5)
Types of FP method used (*n* = 234)	
Injectable	91 (38.9)
Implants	77 (32.9)
Oral pills	44 (18.8)
IUCD	17 (7.3)
Others^a^	5 (2.1)
The periods when they started to use FP methods (*n* = 234)	
< 6 weeks	39 (16.7)
6 week -3 month	103 (44.0)
3–6 month	48 (20.5)
6–9 month	32 (13.7)
9–12 month	12 (5.1)
Started to use FP methods in relation to menses resumption (*n* = 234)	
Before menses resumption	18 (7.7)
In the same week when menses resumed	67 (28.6)
After menses resumed	149 (63.7)
Reasons for not using FP methods (n = 166)	
No return of menses	48 (28.9)
On breastfeeding	37 (22.3)
Partner opposed	28 (16.9)
Fear of side effects	23 (13.9)
Fear of change in breast milk	20 (12.0)
Others^b^	10 (6.0)

^a^Condom and Tubal ligation

^b^Prohibition of religion and want to be pregnant

### The attitude of mothers toward the utilization of modern FP methods

Overall, based on the predetermined criteria, 205 (51.2%) mothers had a favorable attitude towards modern FP methods. Of the mothers, 207 (51.7%) agreed that FP helps the mother to regain her strength before her next baby, and 210 (52.5%) agreed that birth spacing decreases maternal death (Table 4).

**Table 4:** Attitude towards utilization of modern FP methods among mothers who gave birth in the last 12 months at Injibara town, Northwest Ethiopia, 2019, (*n* = 400)

**Variables**	**Strongly disagree**	**Disagree**	**Neutral**	**Agree**	**Strongly agree**
FP helps the mother to regain her strength before her next baby	0(0.0%)	10(2.5%)	33(8.3%)	207(51.7%)	150(37.5%)
Birth spacing decreases maternal death	0(0.0%)	5(1.2%)	18(4.5)	210(52.5%)	167(41.8%)
Birth spacing protects children's death	17(4.2%)	20(5.0%)	51(12.8%)	189(47.2%)	123(30.8%)
Unmarried women can use FP methods	55(13.8%)	106(26.5%)	117(29.2%)	84(21.0%)	38(9.5%)
FP utilization did not affect cultures	64(8.5%)	67(16.8%)	114(28.5%)	118(29.5%)	67(16.8%)
Utilization of PPFP is good for the standard of living	29(7.2%)	45(11.3%)	66(16.5%)	145(36.3)	115(28.7)
Discussing the utilization of PPFP use with the husband is good	9(2.2%)	17(4.2%)	43(10.8%)	209(52.3%)	122(30.5%)
FP utilization helps to strengthen confidence between couples	39(9.8%)	85(21.2%)	109(27.2%)	112(28.0%)	55(13.8%)
Attitude towards FP methods					
Favorable attitude	205 (51.2%)				
Unfavorable attitude	195 (48.8)				

### Factors associated with the utilization of modern PPFP methods during the extended postpartum period

In bivariate analysis: maternal education level, knowledge about modern FP methods, number of ANC visits, counseling about FP during ANC visits and immediately after delivery, previous use of FP methods, having PNC check-up, linkage to FP unit during child immunization, menses, and sexual resumption after birth, discussion about FP methods utilization with a partner, and attitude towards utilization of modern FP methods were candidate variables for multivariable analysis at *P*-value less than 0.2. In the multivariable analysis, secondary and above educational level, having three and above ANC visits, being counseled on FP methods utilization during ANC visits, resumption of menses, linkage to FP unit during child immunization, discussing with the partner regarding FP method utilization, good knowledge of modern FP methods, and getting counseling on the utilization of FP methods immediately after delivery remained significantly associated with utilization of modern PPFP at a *P*-value of less than 0.05.

Mothers who had secondary and above educational levels were 2.65 times more likely to utilize modern PPFP methods [AOR = 2.65, 95% CI = 1.12–6.25] relative to mothers who had no formal education, and mothers who had three and above ANC visits were 2.13 times more likely use modern PPFP methods [AOR = 2.13, 95% CI = 1.15–3.88] than who had less than three ANC visits. Counseling on the utilization of modern FP methods during ANC visits increases the odds of the utilization of modern PPFP methods by 2.33 times [AOR = 2.33, 95% CI = 1.32–4.11] than not counseling on the utilization of FP methods, and postpartum women whose menses was resumed were 2.48 times more likely utilize modern PPFP methods [AOR = 2.48, 95% CI = 1.20–5.13] relative to those whose menses was not returned. Mothers who are linked to the FP unit during the child immunization were 8.80 times more likely to use modern PPFP methods [AOR = 8.80, 95 CI = 4.87–15.88] than those who are not linked to the FP unit. Discussing the utilization of modern FP methods with a partner increased the utilization of modern PPFP methods by 3.01 times [AOR = 3.01, 95% CI = 1.47–6.17] relative to those who have not discussed the utilization of the methods with their partner. Mothers who are counseled on the use of modern FP methods immediately after delivery were 2.16 times more likely to utilize modern PPFP methods [AOR = 2.16, 95% CI = 1.05–4.43] than mothers who are not counseled. Moreover, mothers who have good knowledge of modern FP methods were 2.01 times more likely to use modern PPFP methods [AOR = 2.01, 95% CI = 1.07–3.78] than those who have poor knowledge of modern FP methods (Table 5).

**Table 5:** Logistic regression analysis for the utilization of modernPPFP methods during the extended postpartum period among mothers who gave birth in the last 12 months at Injibara town, Northwest Ethiopia, 2019, (*n* = 400)

**Variables**	**Utilization of modern PPFP methods in the extended postpartum period**		**COR( 95% CI)**	**AOR(95% CI)**	***P*-value**
	**Yes**	**No**			
Maternal educational level					
Had no formal education	20	35	1	1	
Primary education	59	60	1.72 (0.89–3.32)	1.24 (0.50–3.07)	0.646
Secondary and above	155	71	3.82 (2.06–7.08)	**2.65 (1.12–6.25)**	0.026*
Numbers of ANC visits					
Less than three	48	59		1	
≥ Three	186	107	2.14 (1.36–3.35)	**2.13 (1.15–3.88)**	0.016*
Counseling on FP methods during ANC					
No	85	102	1	1	
Yes	149	64	2.79 (1.85–4.21)	**2.33 (1.32- 4.11)**	0.003*
Discussed contraceptive use with a partner					
No	71	118	1		
Yes	155	42	6.13 (3.91–9.62)	**3.01 (1.47–6.17)**	0.003*
Previous use of FP					
No	76	106	1	1	
Yes	158	60	3.67 (2.42- 5.58)	0.78 (0.36–1.65)	0.513
Counseled on FP methods utilization immediately after delivery					
No	41	105	1	1	
Yes	190	59	8.25 (5.18–13.12)	**2.16 (1.05–4.43)**	0.035*
PNC checkup					
No	99	114	1	1	
Yes	135	52	2.99 (1.95 – 4.54)	0.56 (0.27–1.17)	0.124
Linkage to FP unit during child immunization					
No	26	88	1	1	
Yes	180	51	11.95 (6.98–20.43)	**8.80 (4.87–15.88)**	0.001*
Menses return after the childbirth					
No	68	123	1	1	
Yes	166	43	6.98 (4.64 -10.92)	**2.48 (1.20- 5.13)**	0.015*
Resume sexual activities					
No	47	106	1	1	
Yes	187	60	7.03 (4.48 – 11.02)	1.67 (0.84–3.31)	0.142
Attitude towards FP					
Unfavorable Attitude	83	112	1	1	
Favorable Attitude	151	54	3.77 (2.48–5.75)	0.53 (0.27–1.05)	0.069
Knowledge of FP					
Poor knowledge	51	101	1		
Good knowledge	183	65	5.58 (3.59- 8.66)	**2.01 (1.07–3.78)**	0.030*

^*^Significant at a *P*-value of < 0.05

## Discussion

The utilization of modern PPFP methods in the extended postpartum period offers a unique opportunity to ensure the pregnancy is either avoided or postponed until the woman's condition is optimized. This study shows that the prevalence of utilization of PPFP methods among postpartum women was 58.5% [95%, CI: 53.5 – 63%]. Injectables (38.9%) and Implants (32.9%) were the most frequently used methods. This finding is in line with other studies conducted in South Gondar (54.7%) [27], Northwest Ethiopia (60.6%) [30], and Kenya (59.0%) [31]. However, utilization of modern PPFP methods in this study is higher than other studies conducted in Ethiopia like Gozamen district (46.7%) [29], Bahir Dar city (48.8%) [32], Burie District (20.7%) [33], Gondar (45.8%) [22], Dabat (10.3) [34], Debre Berhan (41.6%) [35], Arba Minch town (44%) [36], Aksum (48%) [24], Gida Ayana district Oromia region (45.4%) [37], rural Tigray region 38.3%) [38], Kebribeyah Town, Somali Region, Eastern Ethiopia (12.3%) [26], Durame Town 36.7% [39] and 2016 EDHS secondary data analysis report (23%) [40]. The probable reason for this difference might be the time gap between the studies. As seen over time, the utilization of maternal health care services increased, thus it may help to get information about the advantage of the utilization of modern FP methods.

It was also higher than studies conducted in Nigeria (39.8%) [41], Uganda (28%) [42], Nepal (37%) [43], India (17%) [44], Burundi and Rwanda, 20% and 51% of women utilized PPFP respectively [45]. The difference might be attributed to sociodemographic and reproductive characteristic variation. The marital status of women may have contributed to the differences in the prevalence of utilization of modern PPFP methods. If a woman is married, she may have earlier postpartum sexual contact than those who are not married. For instance, the proportion of married women in our study was over 96.0%, as against 83.6% in the study done in Uganda.

However, this finding was found to be lower compared with studies done in Addis Ababa (80.3%) [17] and the Ganta-Afeshum District Eastern Tigray region (68.1%) [46]. The difference could be due to the presence of variation in service accessibility. It was also lower than studies conducted in Rural Kenya (86.3%) [47] and Ntchisi district hospital Malawi (75%) [48]. The possible reason for this discrepancy might be attributed to the differences in policy, access, and maternal health care service utilization.

In this study, socio-demographic, reproductive characteristics, maternal health care service utilization, and knowledge level of the mothers were significantly associated with the utilization of modern PPFP methods. The odds of utilizing modern PPFP methods during the extended postpartum period were 2.65 times higher for mothers who had secondary and higher educational levels. This finding is in line with other studies [24, 29, 30, 33, 36, 49]. The possible explanation could be educational attainment increases health care service-seeking behavior and a better understanding of the benefits of birth spacing and limited family size using FP methods.

Having three and above ANC visits increased the chance of utilizing modern PPFP methods by 2.13 times. This study is supported by cross-sectional studies conducted in Arba Minch town and Ganta-Afeshum District [36, 46], Kebribeyah Town Somali Region [26], Aroressa District, Southern Ethiopia [50], and India [44]. The possible reason might be that those mothers who had frequent ANC visits may have more exposure to information on the advantage of birth spacing and the complication of early delivery for both mothers and their newborn babies.

In this study, mothers who were counseled about FP method utilization during their ANC visits were 2.33 times more likely to utilize modern PPFP methods than their counterparts. This study is congruent with other studies [24, 26, 30, 50]. This might be because those women who utilize FP methods may be properly counseled by health care providers during their ANC visits about the available methods of FP and the consequences of frequent childbirths.

Mothers who had discussed the utilization of FP methods with their partners were 3.01 times more likely to use modern PPFP methods. This finding is in line with the other studies' findings [29, 30, 33, 51]. Spousal communication is considered an important driving force in the practice of modern PPFP methods [22]. This finding could be explained by the fact that decisions made jointly by agreement of both couples will have better outcomes when compared with the decision made by only one side. The issue of FP is not only the concern of one partner. This finding is also in line with studies conducted in Ghana [52, 53].

Being counseled on the utilization of modern FP methods immediately after delivery increased the chance of utilizing modern PPFP methods by 2.16 times. It is in line with studies conducted in Ethiopia and India [39, 54]. The postpartum period provides a crucial window of opportunity in which to address the unmet need for FP methods for several reasons, including the health benefits of an increased inter-pregnancy interval for mother and child and opportunities for interactions between women and health providers.

Mothers who are linked to the FP unit during their child`s immunization were 8.80 times more likely to use modern PPFP methods. This is in line with studies conducted in Northwest and Southern Ethiopia [30, 55]. The possible explanation might be that child immunization creates a good opportunity for counseling about the advantage of FP utilization, birth space, and raising maternal and child health-related issues. Maternal health care services and regular immunization services are a continuous point of contact to provide information about FP, offer services, and link women to PPFP services [54].

Mothers who had good knowledge of modern FP methods were 2.01 times more likely to use modern PPFP methods. The possible reason might be having good knowledge of FP methods may increase the chance of utilizing modern PPFP methods after delivery to avoid too spaced pregnancy. This finding is congruent with studies conducted in Ethiopia [22, 27, 29].

The resumption of menses has increased the utilization of modern PPFP methods by 2.48 times. This finding is supported by studies conducted in different parts of Ethiopia [22, 27, 29, 30, 35, 50], and by a study done in Malawi [48]. This finding could be justified by postpartum women whose menses have returned after delivery may assume that they are at risk of getting pregnant, so this can encourage them to start postpartum FP methods early. The other probable reason might be those women whose menses have resumed, and at the same time, their sexual activities may resume. Because of this, they may perceive that they are at risk of unintended pregnancy and this, in turn, may motivate them to use FP methods.

### Limitation of the study

Recall bias might be introduced in some of the questions that required women to recall past information.

## Conclusion

In our study, the prevalence of utilization of modern PPFP methods during the extended postpartum period among postpartum women in Injibara town was low compared to the WHO recommendation for postpartum women. Maternal educational status, the number of ANC visits, counseling about FP during ANC visits and immediately after delivery, discussing the utilization of modern PPFP with a partner, resumption of menstruation after delivery, having good knowledge of FP methods, and linkage to FP unit during child immunization were significantly associated with utilization of PPFP methods during the extended postpartum period. Therefore, it is important to strengthen FP counseling during ANC visits, delivery, and child immunization to reduce the missed opportunities for postpartum women to get contraceptive methods. Because this contact point with health care providers is a good entry point or creates an opportunity to provide appropriate information about FP methods and to reduce the missed opportunity for postpartum women to get modern FP methods. Strengthening the empowerment of women by increasing access to getting an education is also a crucial step to increasing the knowledge of women about their health and the services provided in the health facility. It is also needed to advise women to have a bilateral discussion with their husbands regarding their reproductive issues. Moreover, it is also important to encourage women to start using modern FP methods before the resumption of menses to reduce the risk of getting an unintended pregnancy.

## Data Availability

All related data have been presented within the manuscript. The data set supporting the conclusion of this article is available from the corresponding author upon reasonable request.

## Abbreviations

ANC: Antenatal Care
EDHS: Ethiopia Demographic Health Survey
FP: Family Planning
IUCD: Intra-Uterine Contraceptive Device
PNC: Postnatal Care
PPFP: Postpartum Family Planning
WHO: World Health Organization
